# Complete chloroplast genome sequence of an endangered tree species, *Magnolia sieboldii* (Magnoliaceae)

**DOI:** 10.1080/23802359.2018.1532826

**Published:** 2018-10-26

**Authors:** Jianmei Gao, Yu Song, Baojiang Zheng

**Affiliations:** a Center for Integrative Conservation, Xishuangbanna Tropical Botanical Garden, Chinese Academy of Sciences, Menglun, Mengla, Yunnan, China;; b State Key Laboratory of Tree Genetics and Breeding, College of Life Sciences, Northeast Forestry University, Heilongjiang, China

**Keywords:** *Magnolia sieboldii*, endangered species, chloroplast genome, phylogenetic analysis

## Abstract

The characteristic of complete chloroplast (cp) genome of *Magnolia sieboldii*, an endangered species in China, was first sequenced in this study. It showed a typical quadripartite structure with a length of 160,177 bp. It composed of a large single copy (LSC) region of 88,238 bp, a small single copy (SSC) region of 18,763 bp, and a pair of inverted repeat (IR) regions of 26,588 bp. Further maximum parsimony phylogenetic analysis was performed based on 38 complete plastomes from 30 magnoliids and basal angiosperm species. The result supported a close relationship among *M. grandiflora*, *M. officinals*, *M. sieboldii,* and *M. tripetala*.

*Magnolia sieboldii* K. Koch is available most in the northern *Magnolia* species in China, it is distributed in the narrow geography regions and scattered in North China, Japan and North Korea (http://foc.eflora.cn/). It is an attractive horticultural and important economic tree species. It is already listed as a vulnerable species in China Species Red List and protected by the Chinese government as the third kind of rare and endangered plants as well. Here, we determined the complete chloroplast genome sequence of *M. seiboldii*.

Fresh young leaves of *M. seiboldii* were harvested from Tiannv mountain of Fushun County, Liaoning Province, China (41.5°N, 124.3°E). Six grams silica-gel dried young leaves were used for DNA extraction (Doyle and Dickson 1987). The complete cpDNA was sequenced following the method of Yang et al. (2014). The genome of *M. sieboldii* was assembled using BioEdit software. Dual Organellar Genome Annotator (DOGMA) software was used to annotate the gene type of *M. sieboldii* (Wyman et al. 2004). The voucher specimens (Voucher number: HITBC-BRG-SY35347) of *M. sieboldii* were deposited at the Herbarium of Xishuangbanna Tropical Botanical Garden (HITBC), Chinese Academy of Sciences.

The plastome of *M. sieboldii* (MH544144) shows a typical quadripartite structure with a length of 160,177 bp. It composes of a large single-copy (LSC) region of 88,238 bp, a small single copy (SSC) region of 18,763 bp, and a pair of inverted repeat (IR) regions of 26,588 bp. The GC-content of the whole plastome is 39.2%, and in LSC, SSC, and IRs regions are 37.9%, 34.3%, and 43.2%, respectively. The plastome contains a total of 113 genes, including 79 protein-coding genes, 30 tRNA genes, and four rRNA genes.

To determine the phylogenetic location of *M. sieboldii* with respect to the other 29 magnoliids and basal angiosperm species with fully sequenced chloroplast genomes, the complete plastome of *M. sieboldii* was used to reconstruct the phylogenetic relationships. Take the plastome of *Amborella trichopoda* (Amborellaceae, Amborellales; AJ506156) as an out-group, a maximum likelihood analysis based on the GTR + I + G model was performed with RAxML version 8 program using 1000 bootstrap replicates (Darriba et al. 2012; Stamatakis 2014). The phylogenetic tree in Figure 1 revealed that *M. sieboldii* was most closely related to *M. grandiflora*, *M. officinals*, and *M. tripetala*, which was in agreement with previous reports on the relationships among them (Azuma et al. 2001; Nie et al. 2008). Magnoliaceae was strongly supported as monophyletic (ML-BS = 100%), and four well-supported groups were recovered within these *Magnolia* species. In the Magnoliaceae, the basal group (ML-BS = 100%) including the two species of Liriodendron, the second group (ML-BS = 100%) including *M. pyramidata* and *M.dealbata*, the third group (ML-BS = 95%) including *M. grandiflora*, *M. sieboldii*, *M. tripetala*, and *M. officinalis*, and the core group (ML-BS = 95%) including *M. kwangsiensis*, *M. yunnanensis*, *M. sinica*, *M. cathcartii*, *M. odora*, *M. acuminata*, *M. sprengeri*, *M. salicifolia*, *M. biondii*, *M. kobus*, *M. liliiflora*, *M. liliifera,* and *M. denudata*.

**Figure 1.**
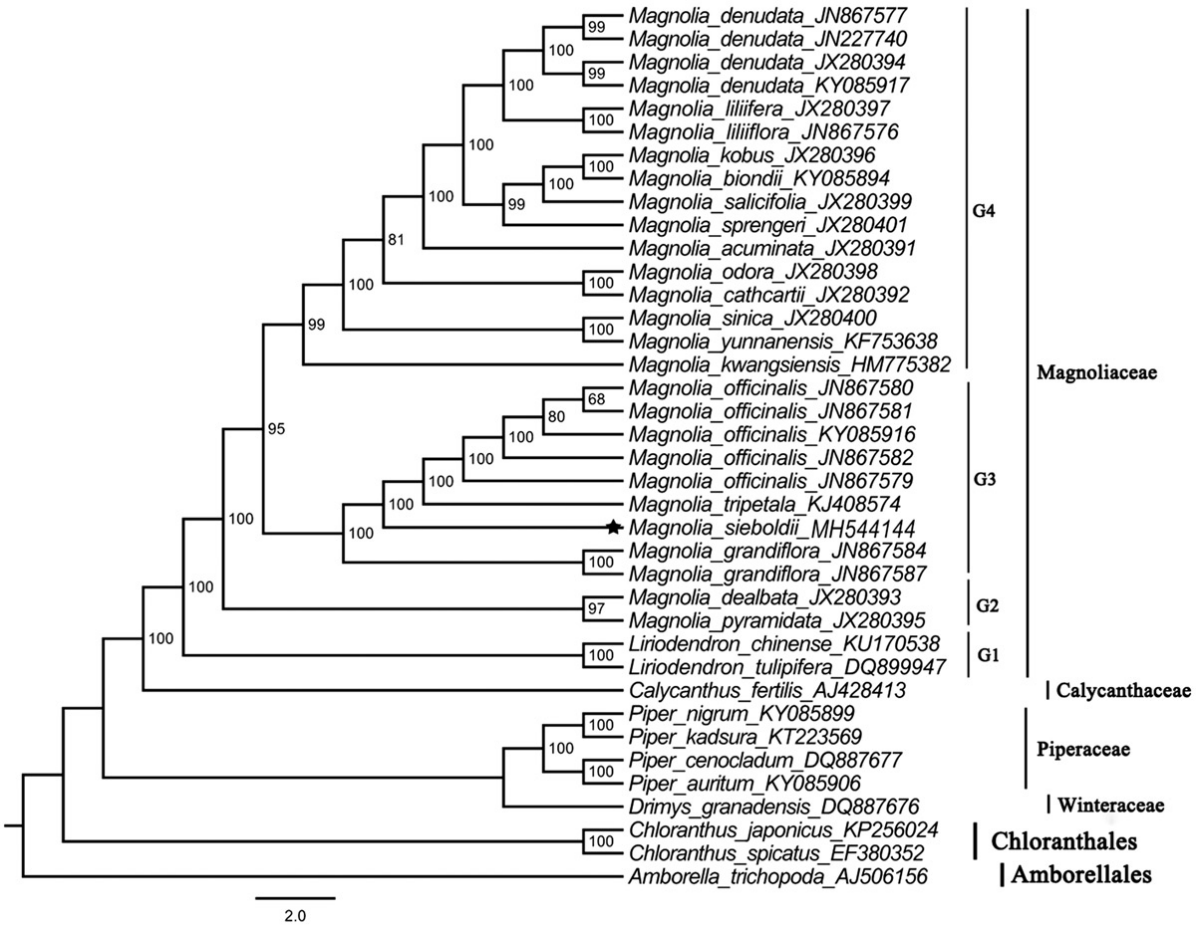
ML phylogenetic tree based on 38 complete chloroplast genome sequences from 30 species.

## Data Availability

The plastome data of the *M. sieboldii* was submitted to Genebank of NCBI. The accession number from Genebank is MH544144.
